# Prescribing of Psychiatric Drugs in Pregnancy: A Review of Current Practice in a Community Mental Healthcare Setting

**DOI:** 10.1192/bjo.2022.444

**Published:** 2022-06-20

**Authors:** Lucy Hickling, Nilamadhab Kar

**Affiliations:** Black Country Healthcare NHS Foundation Trust, Wolverhampton, United Kingdom

## Abstract

**Aims:**

Prescribing of psychotropic medications in pregnancy is still considered a ‘grey area’ in clinical practice. National Institute for Health and Care Excellence (NICE) in the UK suggests that the decisions should be person-specific, considering the risks to both the mother and unborn child, and the patient is supported to make an informed decision. It is important to explore the use of psychotropic medications during pregnancy, or lack of it, and its subsequent impact on maternal mental health. It was intended to review the decisions expectant mothers are making regarding taking psychiatric medications during pregnancy, and the associated clinical outcomes. Their mental capacity for taking decisions was also checked.

**Methods:**

A retrospective audit of case notes of patients (n = 16) known to community psychiatric team (CMHT) and specialist perinatal mental health (PNS) services in Wolverhampton, who notified their pregnancy between December 2020 and December 2021. Demographic and clinical data were collected from the electronic records.

**Results:**

The sample had a mean age of 28.8 ± 6.3 years (range: 19 to 39 years), and 68.8% of them were Caucasian. A wide range of psychiatric diagnoses were present, most (62.5%) had comorbid diagnoses; and 25% had substance use, most commonly cannabis. Mean duration of gestation at the review following notification of pregnancy was 14.5 ± 7.7 (range: 6 to 29) weeks. It was observed that 25% were not taking psychiatric medications prior to pregnancy, 43.8% stopped taking their medication prior to the psychiatric review, most stopping abruptly, and 31.2% had continued their medication. The medications included aripiprazole, olanzapine, quetiapine, venlafaxine, sertraline and promethazine. Following the review, only 18.8% continued their medications. Of the 13 (81.3%) patients who were not taking medications, 9 (69.2%) had adverse mental health outcomes, with 2 (15.4%) patients requiring inpatient care. However, later 8 (61.5%) started taking medications whilst under the care of PNS. All of them had mental capacity to decide regarding their psychiatric treatment at the review.

**Conclusion:**

Most psychiatric patients avoided taking psychotropic medications initially during pregnancy, however, a considerable proportion restarted their medications following review with the perinatal psychiatry team. The majority of patients who did not take medications had negative mental health consequences. It is important to develop an evidence base about the use of psychiatric medications in pregnancy and the associated short and long-term outcomes that may help the quality of information shared with patients.

